# Alien introgression to wheat for food security: functional and nutritional quality for novel products under climate change

**DOI:** 10.3389/fnut.2024.1393357

**Published:** 2024-06-12

**Authors:** Eva Johansson, Yuzhou Lan, Olawale Olalekan, Ramune Kuktaite, Aakash Chawade, Mahbubjon Rahmatov

**Affiliations:** Department of Plant Breeding, The Swedish University of Agricultural Sciences, Lomma, Sweden

**Keywords:** wheat production, nutritional and functional qualities, food security, plant breeding, dietary habit

## Abstract

Crop yield and quality has increased globally during recent decades due to plant breeding, resulting in improved food security. However, climate change and shifts in human dietary habits and preferences display novel pressure on crop production to deliver enough quantity and quality to secure food for future generations. This review paper describes the current state-of-the-art and presents innovative approaches related to alien introgressions into wheat, focusing on aspects related to quality, functional characteristics, nutritional attributes, and development of novel food products. The benefits and opportunities that the novel and traditional plant breeding methods contribute to using alien germplasm in plant breeding are also discussed. In principle, gene introgressions from rye have been the most widely utilized alien gene source for wheat. Furthermore, the incorporation of novel resistance genes toward diseases and pests have been the most transferred type of genes into the wheat genome. The incorporation of novel resistance genes toward diseases and pests into the wheat genome is important in breeding for increased food security. Alien introgressions to wheat from e.g. rye and *Aegilops* spp. have also contributed to improved nutritional and functional quality. Recent studies have shown that introgressions to wheat of genes from chromosome 3 in rye have an impact on both yield, nutritional and functional quality, and quality stability during drought treatment, another character of high importance for food security under climate change scenarios. Additionally, the introgression of alien genes into wheat has the potential to improve the nutritional profiles of future food products, by contributing higher minerals levels or lower levels of anti-nutritional compounds into e.g., plant-based products substituting animal-based food alternatives. To conclude, the present review paper highlights great opportunities and shows a few examples of how food security and functional-nutritional quality in traditional and novel wheat products can be improved by the use of genes from alien sources, such as rye and other relatives to wheat. Novel and upcoming plant breeding methods such as genome-wide association studies, gene editing, genomic selection and speed breeding, have the potential to complement traditional technologies to keep pace with climate change and consumer eating habits.

## 1 Introduction

Wheat is one of the three major crops (together with rice and maize) across the globe as related to production (yield and area) and as a staple (1, 2). Of these three crops, wheat has a specific place as a food crop with a wide range of products, and it is also the most traded of the cereals (3). Wheat thereby contributes largely to food security in various areas worldwide, where wheat is an important or major component of the daily food consumption (1, 2). Due to the high intake of wheat, its nutritional content is of particular importance to secure human health and wellbeing (4–10). Traditionally, wheat has been used for a variety of baked goods, such as breads, chapatis, naan, pancakes, wafers, biscuits, crackers, cakes etc., while pasta and more “whole seed” (e.g., breakfast cereals) are also common wheat-based products (11). Currently, novel wheat-based products are emerging, such as wheat berries (whole grain wheat to be cooked and replace, e.g., rice), seitan (gluten used to replace meat), high moisture meat analogs (HMMA, to replace meat and in these products, wheat proteins are used as building blocks), to make pace with emerging consumer and societal needs (12–15).

As for all other crops, climate change, with increased spells of extreme weather, has a large impact on the production of wheat, affecting both yield and quality (16, 17). Plant breeding has resulted in wheat material adapted to the current prevailing cultivation conditions, which will be altered due to the predicted climate change, eventually resulting in a dramatic decrease in yield (18). The focus of plant breeding has been to increase yield, resistance and end-use quality, saving lines with the best performance in the present climate, which also might have affected the genetic diversity in crops (19–22). Furthermore, plant breeding of wheat might have contributed to a decrease in the nutritional quality, e.g., minerals and phytochemicals, of the wheat, and breeding toward improved bread-making quality may have resulted in an increase in components having a negative effect on human health (allergy, coeliac disease, non-coeliac gluten sensitivity, irritable bowel syndrome) (23). However, recent studies (24, 25) have found no evidence of a lower quality of modern wheat for human health as compared to ancient wheat genotypes. Instead, some studies have indicated the modern processing methods of wheat as a risk factor of their health impact (26). Despite this, the incorporation into adapted wheat of novel genes that contribute yield and quality under climate change conditions, is extremely important (8). Genes can be incorporated into adapted wheat from various sources, including the primary (species easily crossable with wheat), secondary (related species within the same genus), tertiary (wild relatives) gene pools.

The present review paper aims to increase the understanding on how alien (wild relatives of wheat; i.e., from the tertiary gene pool) genes have the potential to contribute to increased food security as well as to improved technological and nutritional quality for traditional and novel wheat products by the use of an increasingly wide array of emerging plant breeding methods. Thus, the paper starts with a description of the current status and needs for improvements, then follows a review of opportunities with the use of alien germplasm in wheat breeding, impact on food security, quality aspects and requirements for novel products, finalizing with an overview of benefits of traditional and novel methodologies for the transfer of alien genes into adapted wheat genotypes in relation to the demands described in the other sections. The conclusion section in the end summarizes the outcomes of the present review.

## 2 Wheat—current contribution to food security, human nutrition and food products, and needs for improvement

Wheat contributes one-fifth of the global food calories and protein to the human population and is therefore, extremely important for global food security (1, 2). Wheat is the main staple crop in temperate zones and is thereby produced in a range of areas across the globe, with major producing areas in Europe, East-, West-, Central- and South- parts of Asia, East-, Central- and North- parts of the USA, South part of Canada, South-Central part of South America and mainly in Ethiopia and South Africa in the African continent. The highest yield (4-5 tons per Ha) is found in Europe and East Asia. Almost 30% of the wheat produced comes from low- and middle-income countries with production units of 1-3 Ha, where 80% of the production is used for food (1). Wheat is also the most traded cereal crop (3). These numbers show that wheat is extremely important for food security, feeding a large part of the human population, traded to meet requirements and needs in many areas, and also an important part for poor and small-scale farmers to supply their daily food intake.

Due to the high intake of wheat, its nutritional content has a high impact on human nutrition. Wheat is known as an important source of a number of health-related components such as protein, vitamins, dietary fiber and phytochemicals (8, 27). As an example, wheat is known to contribute 20% of the daily dietary fiber in the UK, thereby providing protection against cardio-vascular diseases, type 2 diabetes and colorectal cancer (28). Depending on genotypic differences, 1 L of wheat flour is also known to provide 100% of the daily required intake of tocopherols, carotenoids and phenolic compounds (9). Wheat genotypes with high levels of minerals, such as iron and zinc have also been reported (29, 30). The increased consumption of wheat in urban areas, together with an increase in unhealthy dietary patterns by part of the consumers in the Western world (31), calls for an increase in health-promoting components in wheat, one of the major staples worldwide. Furthermore, the predicted climate change will have an impact on both production opportunities and the quality of the wheat produced (32–34), indicating the need for the development of high-yielding and health-promoting wheat genotypes to be grown under new circumstances. Currently, we also see a change in the number of food products developed from wheat, where more traditional ones, such as bread, still have a large share in the market, while novel products, such as vegan and meat analog alternatives, are gaining shares (35, 36). Thus, future wheat genotypes need to fulfill requirements in all these aspects.

## 3 Why alien germplasm for improvements?

Plant breeding is used as a tool to improve the yield, resistance and quality of crops (16). With the current climate change predictions, there is increasing requirements for genes with the ability to contribute to high performance independent of climate-related stresses and diseases (37). Thus, genes from alien germplasm might be an untapped resource of genetic diversity in this novel plant breeding landscape (38). The novel and emerging techniques within plant breeding, such as speed breeding, genomic and genetic tools, phenotyping and genotyping, together with improved opportunities to handle big data, also contribute to the increased opportunities to tap and use alien germplasm in breeding programs (5). Thereby, rare and valuable gene combinations can be searched for in alien wheat lines, and modern plant breeding methods should then be used for their transfer to adapted wheat (39).

The alien resource most commonly used in wheat breeding is rye, from which a number of genes have been transferred (40), while the most commonly utilized *Aegilops* species is *A. tauschii* (41). The transfer of genes from the rye gene pool has resulted in a great increase of the yield potential in wheat (42, 43). Besides the widely explored rye genome, a range of other alien sources have been explored for their opportunities to contribute genes for wheat improvement (40). Thus, Goatgrass (*Aegilops* spp.), i.e., the donor of the D genome to common wheat, has been used to widen the genetic diversity of wheat (44); tall wheatgrass (*Agropyron elongatum*) has been used in wheat breeding through distant hybridization (45); *A. caudata* (46), *Thinopyron bessarabicum* (47), *Amblyopyrum muticum* (48), *Triticum timopheevii* (49), and *T. urartu* (50) are additional alien species evaluated and found to carry potential interesting characters for introgressions into the wheat genome.

## 4 Food security despite climate change—opportunities from the use of alien germplasm

The predicted climate change is expected to have a large impact on food security for the global human population (18, 51). A global temperature increase is part of the prediction, which will result in fewer opportunities to grow crops in areas that are already hot and dry (52), while cold areas might be increasingly beneficial for crop production. However, such a change in cultivation environments will require adaptation of plants to daylight changes and other environmental (e.g., soil) differences between current and future cultivation areas (53). Furthermore, the predicted climate change is expected to result in an increased number of events with extreme weather events (54). As a result, heat, drought and flooding will become more common, and the timing of these events might be more unpredictable (54). Such events of extreme abiotic stress will potentially induce massive yield loss in wheat and other crops (18, 55–57), and especially drought is known as a major threat to food security due to its severe impact on wheat yield (55–57). Thus, for increased food security, alien genetic materials should be used in wheat breeding to fortify tolerance to abiotic stress (32) and to increase disease resistance (38, 58, 59), thereby securing high yield (Table 1).

**Table 1:** Examples of the utilization of alien germplasm to improve (a) quality and yield related traits and (b) resistance traits

Alien species	Genetic input	Phenotypic improvements	References
Quality and yield related improvements			
Rye	Chromosome 1RS	Early root vigor, root length, root biomass, spike density, grain yield, drought tolerance	(32, 60)
Rye	Chromosome 3R	Early drought tolerance, aluminum tolerance, grain protein concentration	(32, 33, 61)
Rye	1R, 7R, 1AL.1RS, 2R, 4R, 5R, 6RS, Sec-1^−^ and Glu-B3^+^	Zn, mixing quality, Arabinoxylan, breadmaking	(62–65)
*Aegilops triuncialis*	Chromosome 5U	Grain softness, biscuit-making quality	(66)
*Aegilops tauschii*	D-genome	Water use efficiency, antioxidant capacity, drought tolerance	(67)
*Aegilops tauschii*	Alleles on D-genome	Yield-related traits	(68)
*Aegilops tauschii*	QTL allele on 4DL	Grain yield	(69)
*Aegilops tauschii*	QTL alleles	Yield-related traits	(70)
	Alleles on 3D and short arm of 7D	Preharvest sprouting resistance, grain size	(71)
Wild emmer wheat	Ancestral QTL alleles	Deep-rooting ability	(72)
*Agropyron elongatum*	Gene *Lr19*	Grain yield, radiation use efficiency	(73)
*Aegilops* spp.	Chromosomes 2S, 2U, 7S, 7U, 1M and 4M, Genome U, S, and D	Zn, Fe, Protein, HMW-GS (Gluten strength, baking quality) and dietary fiber	(74–82)
Synthetic wheat with introgressions from *Triticum carthlicum* × *Aegilops tauschii*	QTLs on 2D, 3A, 4A, 5A, 6A	End use quality in terms of gluten strength parameters	(83)
*Triticum timopheevii*	2A	Grain protein and gluten content	(84)
Disease and pest resistance related improvements			
Rye	Chromosome 1RS	Resistance to strip rust, stem rust, powdery mildew	(43, 58, 85)
Rye	Chromosome 2R	Resistance to Stem rust	(39, 59)
Rye	Chromosome 4R, 5R, 6R	Resistance to stripe rust	(38)
*Leymus mollis*	Gene *TaFBN* and *Ta_Pes_BRCT*	Resistance to stripe rust	(86)
*Leymus racemosus*	Chromosome 7Lr#1	Resistance to Fusarium head blight	(87)
Rye	Chromosome 1R, 2R, 6R	Resistance to Syrian Hessian fly	(38)

Modern wheat usually demonstrates a high yield potential and performance, although, incorporation of alien chromosomes in the wheat genome, i.e., from rye, has in previous studies been shown to increase the yield potential of wheat (42, 43). Thus, 1R and 1RS translocation lines were found with a higher yield than non-translocated lines (43, 88), and 1RS has been reported to contribute to a lower yield reduction compared to non-translocation wheat under drought conditions (89). Furthermore, 1R and 1RS have been found to improve root length and biomass in wheat, benefiting better accessibility to water in deep layers when the surface soil dries up (32, 85, 90). In general, root traits are known to play a key role in drought tolerance (91, 92). Furthermore, chromosome 3R has recently been shown to contribute to drought tolerance in wheat at vegetative growth stages under climate chamber conditions (32).

In addition to the widely explored rye genome, there are other alien species exploited to enhance wheat resilience for dynamic climates, such as the D genome donor goatgrass (*Aegilops* spp.) (44). Several wheat-*aegilops* lines have been reported to hold increased drought (67, 93) and heat tolerance (94) as well as with improved nutrient use efficiency (95). *A. tauschii* have in several studies been found to contribute yield related traits when introgressed into wheat (68, 69). Furthermore, tall wheatgrass (*Agropyron elongatum*) is a perennial crop known to withstand different abiotic stresses. Several wheat cultivars developed through distant hybridization with tall wheatgrass showed increased tolerance to heat, strong light, and hot-dry wind (45). The introgression of the *Lr19* gene from tall wheatgrass has been reported to be responsible for a significant yield increase in wheat (73). A summary of the incorporations of various alien chromosomes into wheat and their effect on wheat improvement is shown in Table 1.

## 5 Functional and nutritional aspects—rooms for improvement by the use of alien germplasm

Quality is a concept describing how good or bad something is, and for wheat it attributes to (i) the end-use quality, (ii) health and wellbeing through consumption, and (iii) how the wheat is produced (96). The present review is focusing on the functional and nutritional aspects, where the functionality is directly related to the end-use quality, while the nutritional aspects are directly connected to health and wellbeing. A few of these characters are encoded by major genes (as described below), but the majority are encoded by quantitative traits loci (QTL) (38). In general, the use of alien introgressions for transfer of improvements of a trait will be more complicated when the trait is determined by QTL instead of major genes. Previous work has shown the success of the transfer of resistance genes encoded by a single major gene, into an adapted genetic background [e.g., (39)]. The fact that introgression of single major genes are easier and more common are depicted in Table 1, showing examples of disease resistance genes transferred as major genes, while introgressions effecting quality is often the result of QTL and impact on several traits.

End-use quality requirements of wheat differs, as wheat is consumed in various forms around the world, e.g., as bread, pasta, noodles, biscuits and pastries, and type of product and processing conditions is largely affecting the functional aspects (16, 97). Several characteristics, such as grain size and hardness, hue, and, to a large extent, protein content and composition, especially of the gluten proteins, determine the functional quality of wheat (8, 98). The gluten proteins, i.e., the glutenins and gliadins, accounting for 80% of the grain proteins in the mature wheat seed, have a significant impact on the bread-making quality as they confer the viscoelastic properties (99, 100). The gluten proteins have been largely studied, they have been found encoded by major genes and the encoding genes have been determined and designated (101). However, the ability of the gluten proteins to polymerize, in the wheat grain and during processing, has also been found a major determinant of the functionality (97) and this character is most likely determined by QTL as it correlates with weather aspects and developmental stages of the wheat plant (102). Several studies have reported that baking and bread-making qualities of wheat flour is affected by the introgression of rye chromosomes into wheat. The most well-known transfer is the 1BL/1RS translocation, known to contribute a yield increase in wheat but also sticky dough performance due to the exchange of some high molecular weight (HMW)-glutenin subunits (GS) encoded on the 1B wheat chromosome to some secalins encoded on the 1R rye chromosome (103). Thus, to retain the functional quality of wheat, introgressions that includes the deletion of major gluten protein genes should be avoided. However, recent studies have shown improved functionality when 1BL/1RS translocation lines where used and gluten protein genes were restored in these (65). Additionally, introgressions from the 3R chromosome has been shown to contribute positively to baking quality stability across various drought treatments (33). Major genes with positive effects on functional quality have been transferred to wheat from other species than rye, e.g., *Aegilops sharonensis* (82), *T. timopheevii* (84), and *Aegilops triuncialis* (104). Additionally, introgressions from *Aegilops* spp., *Agropyron* spp., *Thinopyrum* spp., and *Leymus* spp., contributing major genes or quantitative traits loci (QTL), have been found to contribute positive effects to the functional properties of wheat (38, 105–108).

Nutritional quality of wheat encompasses the content of a range of different elements including protein, micronutrients such as iron (Fe) and zinc (Zn), as well as various vitamins and phytochemicals (8, 9). Micronutrient deficiency (hidden hunger), especially of Fe and Zn, is a major challenge for more than 3 billion people, with women and children mostly affected (7, 109–111). This problem is present in all countries, but with the highest share in developing countries, where the daily diets for most people are cereal crops, often without any additions (111–113). In developed countries nutrient deficiency is instead often related to the intake of a diet high in sugar and fat and low in essential nutrients (114). Considering economics and sustainability, one of the best strategies to address food nutrient deficiency is through biofortification, i.e., increase of nutrient density in food crops (115–118). As for nutritional characters, much less is known than for the gluten proteins in relation to encoding genes and several of them are encoded by QTL and not by major genes. Alien germplasm has been found to harbor a wealth of essential genes associated with levels of these elements (5, 79, 109, 110, 119). The grain protein content (GPC) is known determined by QTL in wheat and also influenced by e.g., starch accumulation in the grain and stressors (abiotic and biotic) during the grain accumulation (8). However, wild wheat relatives have been identified with high GPC values (120, 121). Furthermore, major genes, e.g., *NAM-B1* and *Gpc-2*, have been identified and introgressed into wheat from *T. dicoccoides* (122), and *T. timopheevii* (84), respectively. Also, through the development of synthetic lines, QTL was transferred from *A. tauschii* which contributed increased GPC (105). Elevated Fe and Zn concentrations have been reported in wheat grains with introgressions of 1R, 2R, 3R and 5R (34, 38). Also, in some wheat genotypes bred through hybridization between the wheat landrace Chinese Spring and *Aegilops kotschyi* a significant increase in grain Fe and Zn concentration were reported as a result of the presence of the *A. kotschyi* chromosomes 2S and 7U (77). Similarly, grain Fe and Zn concentration enhancements have been reported in wheat lines carrying genes from chromosome groups 4 and 7 of *A. peregrina* (78). A recent mega QTL analysis (123) have, correspondingly to previous results using recombinant inbred lines [RILs; (124)], identified candidate areas from *T. turgidum* ssp. *dicoccoides*, with genes positively impacting quality traits such as mineral content and abiotic stress tolerance.

Alien germplasms are also reported to have elevated levels of phenolic compounds, beta-glucans and vitamins, compounds that have been reported to have positive effects on human health (80, 107). Dietary fiber, which is an important food component that enhances digestion and regular movement of the bowl has significant variability in alien germplasms that can be exploited and introduced into elite cultivars (75). Similarly, alien material with a high content of bioactive compounds can be selected and used in breeding to increase the nutritional value of wheat (125).

## 6 Quality aspects of novel products and the need for structures—can alien germplasm contribute?

**Figure 1:**
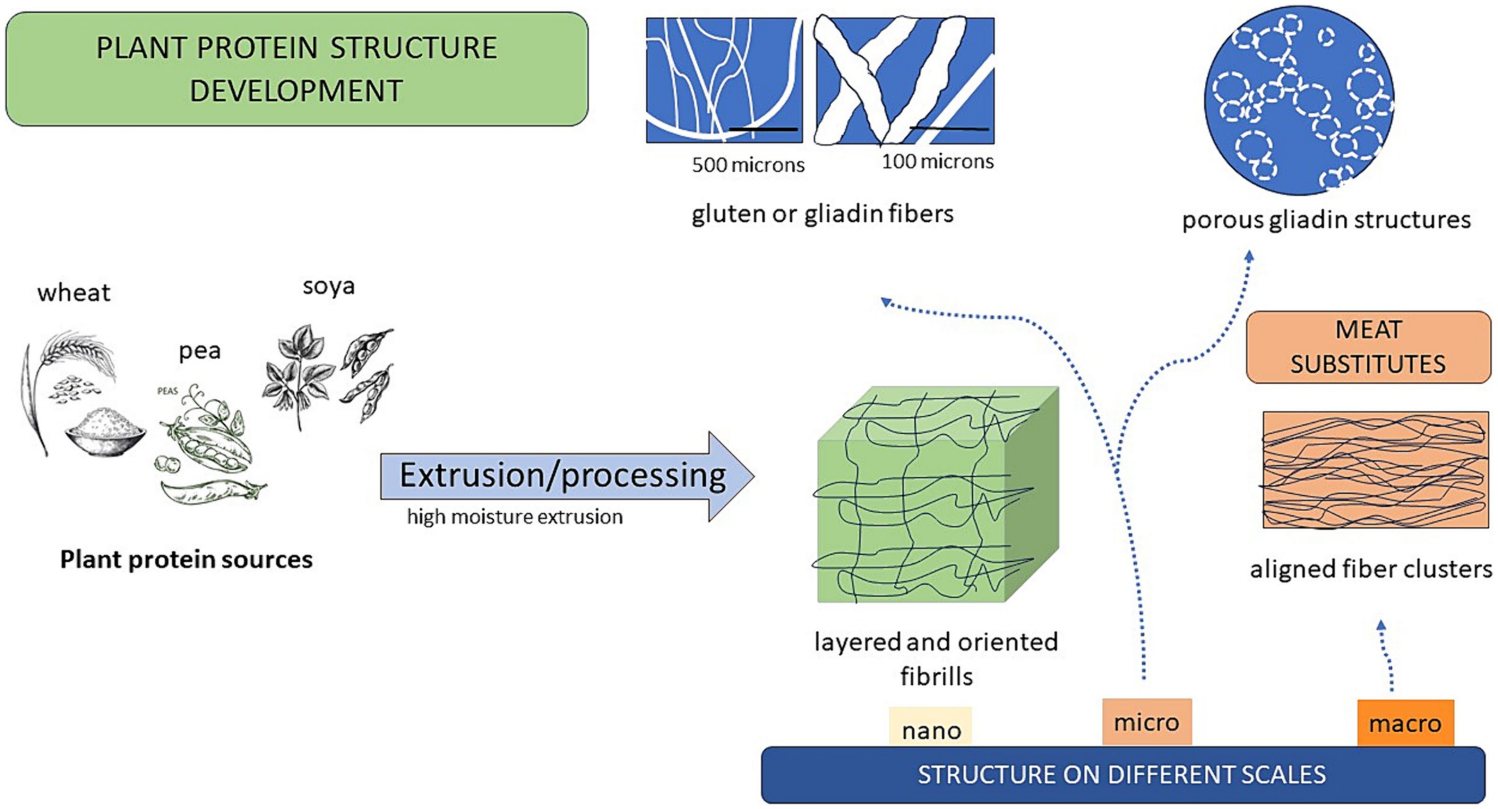
Plant protein fibrous structures at different scales and their suitability for meat substitutes.

Wheat, with its gluten protein (glutenins and gliadins), holds certain quality attributes, which makes it outstanding as a food ingredient and quality contributor. The wheat gluten can easily be texturized e.g., by the use of high moisture extrusion processing (≥40%), resulting in elastic, tough and uniform textures of significance for a variety of food products with different flavors (126). Twin-screw extrusion is commonly used in the process to create novel products such as palatable meat analogs, of value as replacers of animal-based products in the recent food market (127). These products are especially demanded by flexitarians, i.e., consumers who wants to reduce their meat consumption due to environmental or health concerns (128). These consumers often do not want to give up the special taste and texture of meat products. Thus, proteins that are fibrous and highly ordered in elongated structures, along with juiciness, tenderness and attractive flavor, are characters needed in these products (129, 130), as well as a desired nutrient profile. The importance of the fibrous structure formation of plant proteins in meat analogs and their contribution to functionality on different length scales has been highlighted in several studies (131–135) and is summarized in Figure 1. Also, amino acid composition with a high content of essential amino acids for human consumption and a high degree of protein digestibility as well as of other health-promoting components, are of great importance in these products (136).

In addition to wheat, a range of different plant proteins, including soy, pea, peanut and less explored rapeseed (137), rice (138), oat (139), and a few legume crops (mung bean, lupin, faba bean, and chick pea) (126), have been evaluated and are used as a component in texturized meat analogs products. In such products, the matrix is primarily formed through denaturation, aggregation, cross-linking and phase-separation of proteins, and until now, soy, pea and wheat gluten are the most commonly used plant sources (140). The gluten proteins contribute specific and unique qualities, as they have the ability to form network and cross-links on a larger scale than any other plant protein (141). Thus, the gluten proteins of wheat have a special role in the meat analogs as a matrix and structural builder (142). Other sources, such as the legume proteins, or other less explored protein sources such as algal- and fungal-based ingredients (127), and edible insect powder (143) should then be added to contribute nutritional aspects (136).

Alien germplasm has the potential to contribute a range of quality attributes of interest for the production of meat analogs, including improved structural properties of the proteins, increased content of essential amino acids in wheat, increased content of minerals and nutritional elements in wheat, and improved taste performance of the products. Previous studies have indicated a high gluten strength in some alien wheat introgression lines with *Leymus* spp. and 3R (33, 38). Gluten strength is the most important quality criteria for baking and is measured by a range of instruments, among others, by the alveograph (144). Furthermore, multiple introgressions of alien segments into cultivated wheat from *Thinopyrum ponticum*, *Aegilops longissima* and *Triticum aestivum* were found to improve gluten quality characteristics and semolina yellow index in dual-purpose durum wheat (145). As described above, high levels of Fe and Zn and other nutritional components have also been reported in alien germplasm, which could be beneficial in relation to the production of meat analog, to replace the intake of such elements from meat products (34).

## 7 Benefits of traditional and modern breeding methodologies for transfer of alien genes of importance for quality and food security

Alien genes have been transferred into wheat during the 20th century, with several positive outcomes, especially in the area of disease resistance genes (146). Traditionally, such a transfer has included a range of different techniques used, where the inclusion of crosses utilizing genetic material lacking the *Ph1* gene has been an essential part (147). The *Ph1* gene is a key regulator of chromosome pairing during meiosis in wheat, preventing recombination between homoeologous chromosomes, which results in that wheat behaves like a diploid during meiosis despite the crop being a hexaploid (148). Mutations induced in *Ph1* (*ph1b*) allele by radiation allowed increased recombination between the homoeologous chromosomes of wheat and rye (149). Another way of inducing recombination has been the use of nullisomic lines, lacking the chromosome containing the *Ph1* locus (150). However, independent of the methodology used, the procedure has been rather tedious and unprecise, and because of that, the number of commercial varieties containing alien chromosome fragments is rather limited (40). Additionally, the transfer of alien genes into adapted wheat often also results in linkage drag, i.e., the genetic transfer includes a larger piece of the alien chromosome than only the desired gene of interest (151). One of the most well-known examples of such a transfer is the 1BL/1RS translocation that resulted in the variety Petkus, which contributed higher yield and tolerance to abiotic stresses but simultaneously reduced the gluten strength and gave sticky doughs (152, 153). Another example is the transfer of the stem rust resistance gene *Sr22* from *T. boeoticum* to wheat which caused a yield penalty and additional efforts were needed to reduce the linkage drag (152).

However, novel techniques such as whole genome sequencing and platforms with genetic data, as well as high-throughput phenotyping methodologies and large data set handling opportunities and speed breeding techniques have resulted in novel opportunities for a quicker and more specific transfer of alien genes into adapted wheat lines. Thus, recent advances show good opportunities for quick and precise transfer of alien genes using, e.g., genotyping-by-sequencing both for determination of the gene of interest (153), for the development of competitive allele-specific PCR (KASP) markers (154, 155) and for the selection of adapted wheat material containing the transferred gene (39, 156). Thus, the use of these novel technologies will most likely result in increased use of alien germplasm to transfer genes of interest both as related to quality traits (end-use, structure, nutrition) and resistance and yield, thereby targeting food security.

Additionally, precision genome editing, using the CRISPR-Cas9A technique is rapidly becoming popular. In wheat, it has been used to improve drought tolerance (157) and herbicide tolerance (158). As the acceptance of the use of CRISPR-Cas9 increases, it can become the method of choice for mirror alien genes of interest to have in wheat. CRISPR-mediated gene editing can facilitate the replacement of endogenous alleles with desired alien alleles carrying beneficial traits. By introducing precise mutations or sequence modifications using CRISPR, it can be possible to replace target gene variants in the crop genome with those from wild or related species, thereby introducing novel traits or improving agronomic performance. CRISPR systems can be multiplexed to simultaneously target multiple genomic loci, allowing for the introgression of multiple alien genes or alleles in a single transformation event. This enables the rapid introgression of complex trait combinations from wild or exotic germplasm into elite crop varieties, accelerating the breeding process. But to succeed with it, deeper knowledge of the functioning of causative alien genes is needed, followed by introducing similar mutations in the corresponding wheat alleles. CRISPR-Cas9 was used in a study that targeted α-gliadin genes to produce low gluten containing wheat lines with reduced immune reactivity. Upto 35 different genes were mutated in one of the lines and immunoreactivity was reduced by 85% (159). Thus, precision genome editing can become a promising alternative to homoeologous recombination between alien and wheat chromatin. With developments in next generation sequencing and comparative genomics key genes can be identified in the alien genomes to later on mirror those in the wheat genome.

## 8 Conclusion

**Figure 2:**
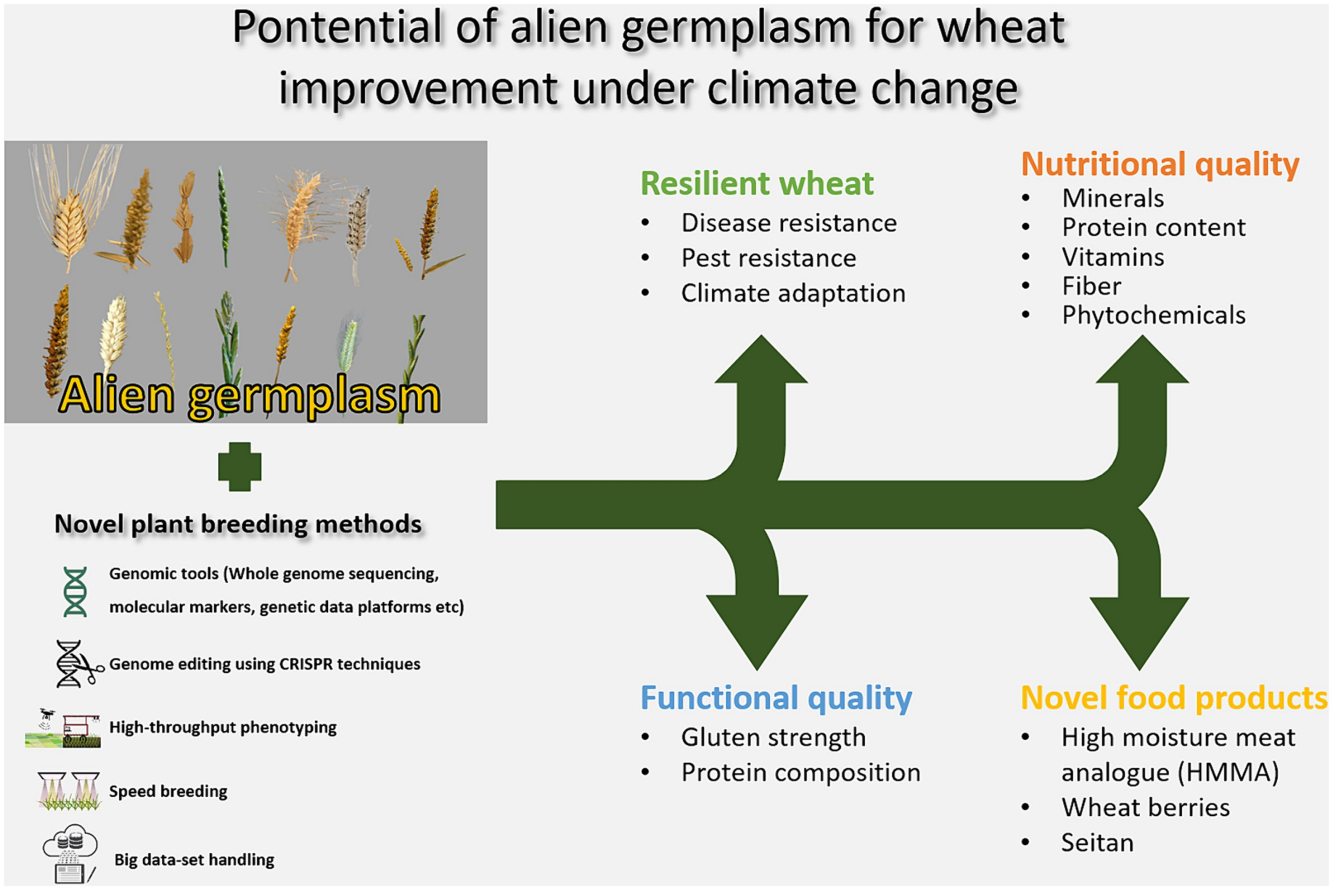
Potential of alien germplasm for wheat improvement under climate change.

The predicted climate change calls for more resilient, stable and robust crops, which is important not least as related to the staple crops such as wheat, feeding a large proportion of the human population. Plant breeding has resulted in a large increase in yield, resistance toward diseases and pests, and quality in crops currently under cultivation, although it has also narrowed the genetic base. Widening of the gene pool in the cultivated crops and the introduction of new genes that allows future cultivation of high yielding and stable cultivars is becoming more important than ever with the growing world population and changes in cultivation environments. Here, alien resources, holding genes contributing to novel resistances toward diseases and pests, tolerances to e.g., heat, drought and flooding, and qualities are becoming increasingly important. Alien genetic material have been shown to have potential to contribute genes targeting improved functional and nutritional qualities in wheat, as summarized in Figure 1. Furthermore, stability and resilience to various climatic conditions have been demonstrated in wheat material containing alien chromosome fragments. Novel eating habits and the transition toward a replacement of animal-based products with plant-based alternatives paves the way for unique quality desires of the plants for these products. The wheat proteins are unique, with the ability to form large polymers through cross-linking of the proteins. Thus, the wheat proteins are needed to form the matrices in many of these novel products. The incorporation of alien genes in wheat may contribute certain qualities of relevance for these new products, both in terms of functionality, e.g., matrices that resembles more meat, and nutrition, e.g., higher mineral levels or lower levels of phytases. Novel plant breeding methods, including whole genome sequencing, platforms with genetic data, high-throughput phenotyping, large data set handling and speed breeding techniques, but also emerging CRISPR techniques, contribute opportunities to use alien resources in wheat breeding in ways that are more precise and with a higher speed (Figure 2).

## Author contributions

EJ: Conceptualization, Data curation, Formal analysis, Funding acquisition, Investigation, Methodology, Project administration, Resources, Software, Supervision, Validation, Visualization, Writing – original draft, Writing – review & editing. YL: Conceptualization, Formal analysis, Investigation, Validation, Visualization, Writing – original draft, Writing – review & editing. OO: Writing – original draft, Formal analysis, Writing – review & editing, Visualization, Investigation, Conceptualization. RK: Conceptualization, Project administration, Supervision, Writing – original draft, Funding acquisition, Investigation, Writing – review & editing, Resources. AC: Writing – original draft, Investigation, Writing – review & editing, Supervision, Conceptualization. MR: Investigation, Conceptualization, Writing – review & editing, Writing – original draft, Supervision.
